# Reduction of Cost and Environmental Impact in the Treatment of Textile Wastewater Using a Combined MBBR-MBR System

**DOI:** 10.3390/membranes11110892

**Published:** 2021-11-19

**Authors:** Xuefei Yang, Víctor López-Grimau

**Affiliations:** 1 Institute of Textile Research and Industrial Cooperation of Terrassa (INTEXTER), Universitat Politècnica de Catalunya, Carrer de Colom 15, 08222 Terrasa, Spain; xuefei.yang@upc.edu; 2 Department of Project and Construction Engineering, Universitat Politècnica de Catalunya, Carrer de Colom 11, 08222 Terrassa, Spain

**Keywords:** textile wastewater, moving bed biofilm reactor, membrane bioreactor, economic feasibility, life cycle assessment, water reuse, decolorizing agent

## Abstract

A hybrid Moving Bed Biofilm Reactor—Membrane Bioreactor (MBBR-MBR) was developed for the treatment of wastewater from a Spanish textile company. Compared with conventional activated sludge (CAS) treatment, the feasibility of this hybrid system to reduce economic and environmental impact on an industrial scale was conducted. The results showed that, technically, the removal efficiency of COD, TSS and color reached 93%, 99% and 85%, respectively. The newly dyed fabrics performed with the treated wastewater were qualified under the standards of the textile industry. Economically, the values of Capital Expenditure (CAPEX) calculated for the hybrid MBBR-MBR system are profitable because of the reduction in Operational Expenditure (OPEX) when compared with CAS treatment, due to the lower effluent discharge tax thanks to the higher quality of the effluent and the decolorizing agent saved. The result of Net Present Value (NPV) and the Internal Rate of Return (IRR) of 18% suggested that MBBR-MBR is financially applicable for implantation into the industrial scale. The MBBR-MBR treatment also showed lower environmental impacts than the CAS process in the life cycle assessment (LCA) study, especially in the category of climate change, thanks to the avoidance of using extra decolorizing agent, a synthetic product based on a triamine.

## 1. Introduction

The textile industry processes a wide variety of fibers: naturals (cotton, wool, etc.), artificial (viscose, acetate, etc.) or synthetic (polyester, acrylic, etc.). Each textile fiber needs a specific processing technology and corresponding sizing agents, dyes, and auxiliaries [1]. The dyeing and finishing processes of the textile industry involve the generation of large volumes of wastewater. The composition of textile wastewater is very complex, and it is often characterized by variable pH, high concentrations of Chemical Oxygen Demand (COD), high turbidity, problems of color and limited biodegradability due to the dyes remaining in the wastewater [2]. The chemical stability and low biodegradability of compounds like dyes in the wastewater especially cause significant environmental concerns [3,4]. In response to such a complex type of wastewater, researchers and industries have developed various treatment processes, such as physico-chemical (coagulation–flocculation, adsorption and filtration), biological technologies and combined treatment processes [5,6,7]. Compared with physico-chemical methods, biological processes are more environmentally friendly because of the complete degradation of contaminants without producing secondary pollutants [8]. In contrast, conventional biological processes by conventional activated sludge (CAS) are not able to properly eliminate the color of textile effluents and they require the application a tertiary treatment for color removal in order to accomplish current regulations [4]. In Spain, the discharge of industrial wastewater to public sanitation systems must comply with the limits established by the local and regional authorities, which manage the treatment facilities [9]. In addition, companies must pay a discharge tax to cover the costs of public sanitation infrastructures. This discharge tax depends on the wastewater volume and the pollutant load [10]. To lower the discharge tax, industries have to increase the efficiency of their wastewater treatment systems, in order to obtain effluents of higher quality that can be reused in the production process.

Among different advanced biological treatments, Membrane Bioreactor (MBR), as a promising process combining biological treatment and membrane filtration, has been increasingly applied for industrial wastewater treatment, including the textile sector [11]. The MBR process has shown several advantages over CAS treatment, such as small footprint, stable effluent quality, high tolerance to high concentrations of organic matters, and lower sludge production [12,13]. Due to the benefits of MBR over CAS reflected in better effluent quality, no additional chemical products being needed and less sludge production, the MBR process has been proved in Life Cycle Assessment (LCA) studies to be a more eco-friendly option, especially in environmental impact related to global warming potential, abiotic depletion and acidification [14,15,16]. In previous techo-economic research, MBR showed a higher cost due to the large energy consumption, but the fact that an MBR plant can reduce the Hydraulic Retention Time (HRT) compensates for the higher power expense [17,18]. However, even though MBR can efficiently treat wastewater with a higher organic load than that of the CAS process, the concentration of biomass is limited in practical applications to avoid affecting the oxygen transfer coefficient [19].

Another biological treatment that has been attracting more and more attention in textile wastewater treatment in recent years is the Moving Bed Biofilm Reactor (MBBR), thanks to its ability to withstand a much higher biomass concentration [20,21,22]. Due to the large number of biofilms fixed on the carriers, the biomass concentration of MBBR can be higher than that of CAS [23]. Previous studies have shown that MBBR can effectively remove COD, but its ability to remove color is limited because of its incompleteness of sludge decantation [24,25]. In practical applications, it is essential to add coagulant to obtain a well-clarified effluent and, in many cases, extra decolorizing agent needs to be used to improve the removal of color [26]. From the economic and environmental perspective, these additional products generate extra cost and result in environmental impacts. For example, most of the decolorizing agents used are quaternary ammonium salt [27], which has a high impact on the toxicity category [14].

A hybrid MBBR-MBR system will be able to improve the sludge decantation of the MBBR system by way of the MBR membrane filtration. Moreover, since a part of the biofilm is formed on the MBBR carriers, the oxygen transfer coefficient will not be influenced, allowing energy saving for aeration. Two previous studies have been published on the performance of biofilm-membrane filtration with complex processes, including anaerobic and aerobic tanks, in the wastewater treatment of the textile industry, showing that this treatment is viable for treating textile wastewater [28,29]. With the effective treatment of MBBR-MBR, the effluent does not need to add a coagulant or decolorizing agent, notably reducing the environmental impact. It should be noted that the previous studies used separate tanks for MBBR and MBR. It could be an attractive option to keep the MBBR and MBR process within one reactor to maximize the advantages of both systems. To the best of our knowledge, no studies have been performed on the reuse of treated textile wastewater by MBBR-MBR. Economically, water reuse in the textile industry will allow particularly important savings, not only for the water cost as a consumer but also for the environmental tax.

In this study, a hybrid system, MBBR-MBR, was studied in the treatment of wastewater from a local textile industry. The study centered on the treating efficiency of eliminating organic compounds, suspended solids and color. Subsequently, new dyeing processes were performed with the treated water to assess the viability of water recovery. The objectives of this study are to evaluate the economic and environmental feasibility of the implementation of the hybrid MBBR-MBR on an industrial scale according to the results of its treating efficiency on which, to our knowledge, no such research has been done. The feasibility analysis of the MBBR-MBR system is based on the comparison with the results of the CAS system of the textile industry that provided us wastewater for this study.

Capital Expenditures (CAPEX) and Operational Expenditures (OPEX) of the MBBR-MBR system are calculated. OPEX are compared with those calculated for the current CAS system. The consumption of electricity and decolorizing agent are considered, as well as the discharge taxes derived from the different efficiency of the two systems. The Net Present Value (NPV) and the Internal Rate of Return (IRR) of MBBR-MBR are also calculated, taking into account that the savings derived from water reuse that entails a reduction in acquisition costs and wastewater discharge.

From the environmental point of view, an LCA study is carried out, comparing the environmental impacts of the combined MBBR-MBR system with respect to the CAS system. These impacts come from the electrical consumption of the two treatments and the consumption of decolorizing agent that is essential in CAS, while the MBBR-MBR system avoids the use of this synthetic product based on a triamine.

## 2. Materials and Methods

### 2.1. Caracterization of Textile Wastewater

The wastewater for the input of the MBBR-MBR reactor comes from a Catalan textile finishing industry, Acabats del Bages, S.A. (Monistrol de Montserrat, Spain). The wastewater was obtained from the outlet of its homogenization tank. Table 1 presents the average values of the main pollutant parameters of the wastewater.

### 2.2. Reactor Description

**Figure 1:**
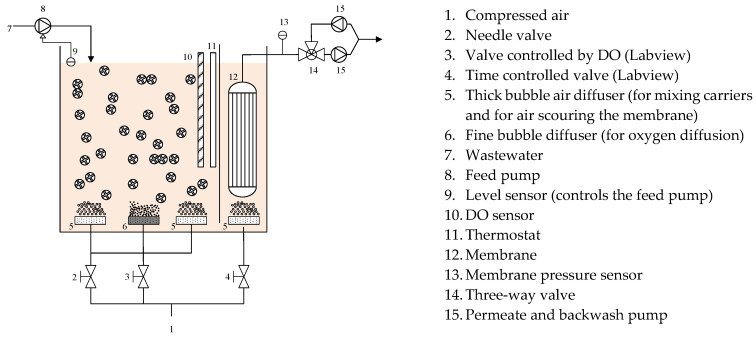
Diagram of the MBBR-MBR pilot plant.

An MBBR-MBR pilot plant was designed and built to determine the efficiency of the combination of moving bed biofilm and membranes technologies on the treatment of textile wastewater. The diagram of the hybrid MBBR-MBR reactor is shown in Figure 1. The effective volume of the reactor is 110 L. The reactor consists of two parts connected at the bottom: MBBR tank (79 L) and MBR tank (31 L). The components of the reactor are presented in Figure 1.

The carriers used in the study are BIOFILL C-2 plastic carriers (BIO-FIL, Barcelona, Spain). The filling ratio of the carriers in the MBBR tank is 25 vol.%. The detailed information is shown in Table 2.

The overall operation of the treatment was 222 days. The ultrafiltration membrane was installed in the MBR tank after the growth of biofilm on the carriers of MBBR was stabled. The flow rate was raised gradually to maintain a steady status of the sludge. At this point, the flow rate was fixed at 4.5 L/h. The period of filtration and backwashing was set at 15 min and 30 s, respectively. The membrane used was a MOTIMO BT01 hollow fiber flat plat membrane (MOTIMO Membrane Technology Co., Ltd. (Tianjin, China)). Characteristics of the UF membrane are included in Table 3.

### 2.3. New Dyeing Processes Reusing the Treated Water

The wastewater treated by the MBBR-MBR process was reused in new dyeing processes in a laboratory dyeing machine Ti-Color (Prato, Italy). Dyeing tests were performed at the following conditions: 10 g of cotton fabric was mixed at a liquor ratio of 1/10 with the dyestuff at a dye concentration 3% o.w.f (on the weight of fiber). Three commercial reactive dyes: Yellow Procion HEXL, Crimson Procion HEXL and Navy Procion HEXL, were supplied by Dystar Inc. 60 g/L of NaCl were included as a dyeing electrolyte. The dyeing process started at 50 °C for 15 min, then the temperature was raised to 80 °C at a gradient of 1.4 °C/min. After 30 min at 80 °C, 20 g/L of Na_2_CO_3_ was added as alkali. Finally, the dyeing lasted for 60 min more. All the experiments were run in triplicate.

After the dyeing process, a washing process was performed to eliminate the unfixed dyes from the fabric. This process consists of nine successive washes performed at a liquor ratio of 1/10.

### 2.4. Analytical Methods

During this study, the treating efficiency of the combined MBBR-MBR system was studied by monitoring the removal of COD, TSS and Color, according to the Standard Methods 23rd edition [30]. TKN, TP and conductivity were also determined, as these parameters are also included in the calculation of the discharge tax.

The performance of the water reusing study was determined following the Standard UNE-EN ISO 105-J03 by comparing color differences between the dyed fabrics made with treated water and the reference made with softened tap water [31]. The calculation of the total color differences (DE_CMC(2:1)_) used Equation (1), considering differences in three parameters: lightness (DL_CMC_), chroma (DC_CMC_), and Hue (DH_CMC_).

DE_CMC(2:1)_ = [(DL_CMC_)^2^ + (DC_CMC_)^2^ + (DH_CMC_)^2^]^1/2^ (1)

A spectrophotometer, MINOLTA CM 3600d (Osaka, Japan), was used for the determination of (DE_CMC(2:1)_) following the Standard illuminant D65/10°. In general, the acceptance limit for color differences in the textile industry is one unit (DE _CMC (2:1)_ ≤ 1). This criterion is widely used in dyeing quality control to compare the color differences between two fabric samples [32].

### 2.5. Economical Analysis

The economic analysis of capital expenditures (CAPEX) and operational expenditures (OPEX) for the MBBR-MBR system is determined by considering individual cost contributions to the treating process. The results are compared with the values of CAPEX and OPEX of the current system applied by the textile company to treat its wastewater. The factory has a conventional biological activated sludge system (CAS). A CAPEX of zero is assigned to the CAS system.

Additionally, the financial feasibility analysis was conducted by examining the Net Present Value (NPV) and the Internal Rate of Return (IRR). The calculation of NPV and IRR is taken into consideration of the investment payback period as 15 years. NPV is the summation of the present value of the net income obtained by folding the income and cost flow back to the starting point of the period. NPV is calculated using the following Equation (2):

(2) $$NPV=\sum_{t=1}^{n}\frac{s}{{\left(1+i\right)}^{t}},$$

in which “*s*” represents the cash flow, “*i*” is the interest rate and “*t*” is the life cycle of investment project [33].

IRR is the interest rate when the cumulative NPV is zero. This IRR means the rate of the largest currency devaluation that the project can withstand. It is also calculated using Equation (2).

### 2.6. Environmental Impact Analysis

In order to evaluate the sustainability and the environmental impact of the hybrid MBBR-MBR system, compared to the existing CAS process, a life cycle assessment (LCA) is performed according to the standard ISO 14040 [34]. Simapro is used with the database Ecoinvent 3.1. The methodologies for the calculation of environmental impact are the ReCiPe, midpoint and endpoint approaches, and the Hierarchist perspective. The functional unit (FU) is set for “1 m^3^ of treated effluent”. The information used in the LCA analysis is taken from the experimental results of the MBBR-MBR pilot plant. In the case of CAS treatment, the system inputs and outputs data are provided by the company.

## 3. Results and Discussion

### 3.1. Efficiency of the MBBR-MBR

The treating performance of the hybrid system was discussed in our previous technical study [35]. Throughout the experiment, the wastewater inlet had an average concentration of COD of 2000 mg/L. The MBBR-MBR treatment achieved an average removal of 93% of COD, with an HRT of 1 day. The removal rate maintained stable although the COD values of influent fluctuated greatly, thanks to the strong resistance of MBBR to shock organic loading [36]. The hybrid MBBR-MBR treatment achieved halving the HRT (2 days) of the actual CAS treatment of the textile industry. Other studies of CAS treatment set a similar HRT within 2 days to achieve the optimum removal efficiency of the organic load in the textile water [37,38]. The decrease of HRT allows significant space-saving or an improvement in the treating capacity. The average membrane pressure for the filtration was 15 kPa, which is much lower than the maximum pressure (80 kPa) that the membrane can withstand, showing that the filtration and backwash was stable, and no membrane fouling was observed during the operation, thanks to the combination with MBBR.

Color of the influent varied between 300 and 1000 mg Pt-co/L during the experiment. The average color removal efficiency was increased to 85% when the treatment was stable. This level of discoloration makes it possible to comply with emission restrictions, which stipulate that no color will appear in samples diluted by 1/30 [9], which allowed the saving on the addition of the decolorizing agent that generally results in significant environmental impact.

TSS removal rate was up to 99%. The MBR part of the hybrid system can produce highly clarified water without the need to add the coagulation products normally required by conventional MBBR. Generally, the use of coagulation agents generates high environmental impact [38].

In the previous study of CAS treating the same wastewater, the average removal rate of COD, color and TSS was 83%, 55% and 66%, respectively [39]. The comparison of the treating efficiency of the hybrid MBBR-MBR system and the CAS system is shown in Table 4. The color removal of 55% of the CAS system was insufficient to comply with current legislation and decolorizing agent must be added, while no decolorizing agent was needed for MBBR-MBR treatment. Compared to the previous study of CAS, MBBR-MBBR obtained a better effluent quality with halved HRT. The elimination of nitrogen and phosphorus was completed in both systems because, generally, textile wastewater does not contain a high concentration of nutrients and, after the biological treatment, the concentrations of TN and TP were very low. These experimental results of the present study and the previous study are used to calculate the economic costs and environmental impacts of the LCA study.

### 3.2. Reuse of the Treated Water

Treated water obtained after the MBBR-MBR process was reused in a new dyeing process. Normally, the evaporation and the adsorption of water into the fiber causes 30% of the water loss during textile production. The quality of the dyes with 100% reused water (ideal reuse) was analyzed to evaluate the possibility of achieving a fully circular dyeing process; however, it would only represent 70% of the input water [40]. Our previous work studied the reuse of the treated water by the hybrid MBBR-MBR, applying tone changes indicating the color differences and the reuse of salt [35]. A new dyeing process was performed with softened tap water as the reference. The color differences of dyed fabrics with treated water and reference fabrics are shown in Table 5. As shown in the table, the DE_CMC(2:1)_ value of Crimson Procion HEXL and Navy Procion HEXL were below 1 within the acceptable range. DE_CMC(2:1)_ value of Yellow Procion HEXL was 1.04, which is on the acceptance limit.

These low color differences are due to the high quality of the water treated by the MBBR-MBR System, which conveniently removed organic material and color. The residual organic matter makes it difficult to fix the new colorant in the reuse processes, and the presence of residual colorants imply changes in hue [14].

### 3.3. Economic Analysis of the Hybrid System

The local textile industry from where the wastewater was taken produces 222,700 m^3^ of wastewater annually. A Conventional Activated Sludge (CAS) system is the current wastewater treatment of the industry, and the daily treatment flow is 920 m^3^/d with HRT of 2 days.

#### 3.3.1. Capital Expenditures (CAPEX)

The CAPEX of the CAS system was taken as the reference (0 €) in the economic analysis. The CAPEX of the MBBR-MBR treatment was added to the reference directly.

For the MBBR part, the expenditure on the carriers with the filling ratio of 25 vol.% (96,250 €) has been taken as the CAPEX estimation according to the suppliers’ information. For the MBR part, the membrane and the installation fees (366,153 €) have been considered for the CAPEX estimation according to a previous study about the cost of a small MBR [41]. In total, the CAPEX of the hybrid system is 462,403 €.

#### 3.3.2. Operational Expenditures (OPEX)

Energy Consumption, information about the decolorizing agent, and the environmental tax generated due to wastewater discharge and sludge production were collected to calculate the operational expenditures (OPEX) of the MBBR-MBR system.

Moreover, the membrane replacement accounted for 2.4% of the energy cost, the maintenance and renovation accounted for 19.5% of the energy expenditure [42] and the average lifetime of the UF membrane was considered to be 10 years. MBBR-MBR can resist a higher organic load with more extended sludge retention time (SRT) than the CAS system, which produced less sludge after the treatment and consequently reduces the frequency of sludge disposal [43]. Throughout the treatment of MBBR-MBR, sludge concentration did not outdo the tolerance limit of the membrane. The production of sludge is estimated based on the increase rate of biomass concentration and the tolerance limit of the membrane.

The OPEX values of a CAS plant in our previous study [39] are listed for the comparison with MBBR-MBR. The detailed OPEX calculation of the existing CAS plant and the MBBR-MBR plant is demonstrated in Table 6 and Table 7, respectively.

Regarding the consumption section, MBBR-MBR had higher electricity consumption (0.21 €/m^3^) because it required more electricity to operate and to maintain the membrane filtration. However, CAS operation cost more in the consumption section (0.55 €/m^3^) due to the use of the decolorizing agent. The decolorizing agent was not required for MBBR-MBR because it achieved the color removal requirement.

In regard to environmental tax, thanks to the great performance of MBBR-MBR treatment on organic matter, TSS removal and sludge generation, it had a lower expense (0.35 €/m^3^) than the expense of CAS (0.86 €/m^3^).

Adding the membrane replacement, maintenance and repair cost, the total OPEX of MBBR-MBR was 0.61 €/m^3^, while CAS is more than twice as expensive to operate with the total OPEX of 1.41 €/m^3^.

Concerning the operation cost of the hybrid MBBR-MBR system, which is much lower than CAS, the initial investment CAPEX is profitable for an industrial plant of wastewater treatment. To confirm that, the economic feasibility is studied in the next section.

#### 3.3.3. Evaluation of the Economic Feasibility (NPV and IRR)

The CAPEX and OPEX of the MBBR-MBR system have been commented on in the above sections. The values of expenditures refer to year zero and have been re-adjusted at a rate of 1.4% yearly in the following years. This rate is taken from the average value of Spain’s inflation target in the next five years [47]. Furthermore, due to wastewater reuse and the pollutant reduction in the wastewater, discharge revenues and some costs being avoided could be achieved. By MBBR-MBR treatment, it was demonstrated that water recovery could reach approximately 70%. The water recovery will allow savings in water consumption cost and discharge tax. The textile industry with CAS treatment pays 0.56 € for each m^3^ of water used [48], and has also been paying 0.86 € for each m^3^ of wastewater discharged. Considering the daily treatment flow of 920 m^3^/d, it was deduced that 644 m^3^ of water could be recovered, and 644 m^3^ of water was not discharged daily. Therefore, the avoided cost of water consumption is 360.64 € daily and the avoided cost of water discharge is 553.84 € daily.

With all these cost data, the assessment of cash flow for 15 years is presented in Table 8.

In terms of considering the NPV for the alternatives, the discount rate was set to 10%. Additionally, as described in the cash flow calculation, the economic life of the MBBR-MBR system was taken as 15 years. By using Equation (2), NPV was calculated. As can be observed, the amortization of the investments will occur in 2 years, where the NPV will be in the order of 85,457 € and for 15 years of operation, the NPV value of the MBBR-MBR system is 193,990 €.

The internal rate of return (IRR) is an evaluation method for investment that finds out the potential rate of return of an asset. When IRR is discounted, the project’s NPV will be zero. It can also be understood as the expected rate of return of a project [49]. IRR is calculated assuming the value of NPV to be zero using the same Equation (2). When a project has a high IRR value, then it can be concluded that the project has high financial feasibility. However, if the IRR value is lower than the discount rate, the application of the system would be unappealing. The IRR value calculated for the MBBR-MBR system is 18%, which is higher than the discount rate assumed (10%), denoting economic feasibility.

Both NPV value and IRR suggest that the MBBR-MBR system is financially applicable for the implantation into industrial scale.

### 3.4. LCA Analysis

#### 3.4.1. Inventory Results

The inventory results of the MBBR-MBR treatment are shown in Table 9. All information is related to the functional unit (1 m^3^ treated water).

#### 3.4.2. Environmental Impact Evaluation

The environmental impact generated by MBBR-MBR treatment conforming to the LCA results is analyzed with an endpoint approach, and the results of MBBR-MBBR treatment are compared with the CAS treatment of our previous study, using the same criteria with reference to their total environmental impact [39].

The results of the environmental impact evaluation are shown in milipoints (mPt) and the differences between categories could be compared. The environmental impact of the MBBR-MBR process was compared with the environmental impact of CAS. The comparison of environmental impacts is demonstrated in Table 10.

During the MBBR-MBR operation, as can be observed, no decolorizing agent was added because the treatment achieved the color removal requirement. The consumption of electricity throughout the operation accounted for the total environmental impact. The results demonstrate that less impact was generated on the Ecosystem; at the same time, the major impacts occurred on Resources and Human Health.

As shown, the MBBR-MBR treatment had a lower impact on all categories, comparing with CAS treatment. Although, according to Table 6 and Table 7, MBBR-MBR had a slightly higher energy consumption of 1.12 kWh/m^3^ compared with 0.96 kWh/m^3^ of CAS, the endpoint results have demonstrated that avoiding the use of decolorizing agent fully compensated for the environmental impact due to higher energy consumption.

**Figure 2:**
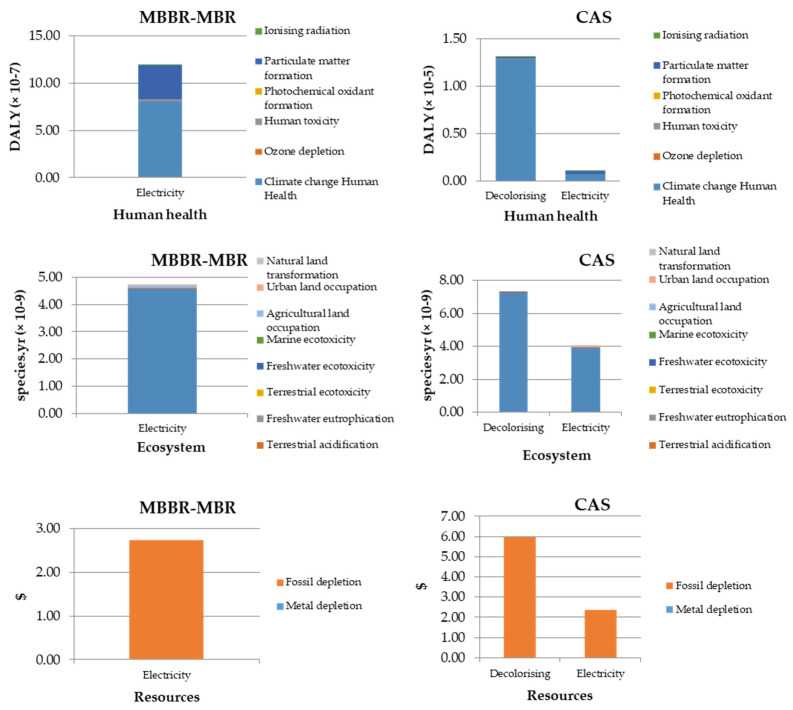
Comparative analysis of the effect of electricity and decolorizing agent consumption on the impacted categories of MBBR-MBR and CAS.

The environmental impacts generated from the MBBR-MBR and CAS treatment are compared in Figure 2, on the specific categories related to Human Health, Ecosystem, and Resources.

Regarding MBBR-MBR treatment, the main factors affecting Human Health due to electricity consumption are Climate Change, Human Health and Particulate Matter formation categories. Meanwhile, the major impact on the ecosystem is attributed to climate change, while agricultural land, terrestrial ecotoxicity, natural land transformation, urban land occupation, and terrestrial acidification had a lower impact on the Ecosystem category. This is due to the composition of the Spanish electricity mix, which in 2020 had a 45.5% electricity generation from renewable sources [50]. In addition, the main impact on Resources came from the Fossil depletion category, while the Metal depletion category had almost no impact.

At the same time, similar results can be observed with CAS treatment; the main environmental impact generated by energy and decolorizing agent consumption related to Human Health and Ecosystem is the Climate change ecosystem. It should be noted that, although the hybrid MBBR-MBR system had higher energy consumption due to the filtration process, no major differences were produced in the related environmental impacts of MBBR-MBR and CAS treatment. In addition, the use of a decolorizing agent in CAS treatment caused many more impacts on all three categories, especially on Human Health. The reason that the consumption of decolorizing agent has a great impact on the category of Climate Change is that the decolorizing agent is DTPA, a triamine. This synthetic product is produced from the alkylation of ammonia. Ammonia production in Europe has an emission factor of 2104 t CO_2_/t NH_3_. Ammonia is synthesized from hydrogen produced by reforming natural gas [51].

While the endpoint methods are useful for decision-making because they can compare results in points, then midpoint analysis can help identify issues of specific environmental concern [52]. The results of the midpoint assessment of MBBR-MBR were also compared with the previous CAS study, shown in Table 11. MBBR-MBR demonstrated more environmental advantages since its impacts were the lower in all the categories, especially in Climate Change, Human Health, Marine eutrophication, Terrestrial ecotoxicity, Freshwater ecotoxicity and Marine ecotoxicity, thanks to the high quality of the effluent treated by MBBR-MBR and the avoidance of using extra decolorizing agent.

## 4. Conclusions

The experimental study of a hybrid MBBR-MBR showed the efficient removal of COD (93%), color (85%) and TSS (99%) with 1 day of HRT. The HRT reduction of 50% for the application of industrial scale is very attractive, resulting in space and energy saving. Additionally, 100% of the water treated by the MBBR-MBR system achieved new dyeings, representing 70% of the textile industry’s water consumption in the dyeing process.

The value of CAPEX calculated for the hybrid MBBR-MBR system is 462,403 € and the increase in CAPEX is profitable due to the reduction in OPEX, which results in lower taxes and savings in decolorizing agent with regard to a small increase in electricity consumption. Additionally, the NPV and IRR study shows that water reuse after the treatment played an important role leading to the cost saving of water consumption and discharge tax. The 18% of IRR calculated demonstrated that MBBR-MBR has great economic feasibility in industrial-scale textile wastewater treatment.

Regarding the LCA study, because of the high efficiency in color removal, the hybrid MBBR-MBR system did not require the addition of decolorizing agent, which is a triamine that has a significant impact on the environment. Therefore, the results suggested that MBBR-MBR treatment generated a much lower environmental impact than CAS treatment.

## Figures and Tables

**Table 1:** Characteristics of the wastewater.

Parameter	Unit	Value
pH	–	8.44 ± 0.54
conductivity	mS/cm	5.15 ± 0.47
Chemical Oxygen Demand (COD)	mg/L	1996 ± 440
5 days Biological Oxygen Demand (BOD_5_)	mg/L	403 ± 88
Total Suspended Solids (TSS)	mg/L	940 ± 121
Total Kjeldahl Nitrogen (TKN)	mg/L	55 ± 21
Total Phosphorus (TP)	mg/L	10 ± 2
Color	mg Pt-Co/L	700 ± 234

**Table 2:** Carriers characteristics of the MBBR system.

Material	Polypropylene (PP)/Polyethylene (PE)
Specific Surface (m^2^/m^3^)	590
Free volume	90%
Carrier diameter (mm)	25
Weight per piece (g)	2.1
Carrier density (kg/m^3^)	<1

**Table 3:** UF Membrane characteristics of the MBR system.

Module	UF BT01 MOTIMO
Configuration	hollow fiber flat plat membrane
Membrane surface (m^2^)	1
Material	polyvinylidene difluoride (PVDF)
Pore size (µm)	0.03
Maximum TMP (kPa)	80
Operating TMP (kPa)	10–60

**Table 4:** Comparison of the treating efficiency of the hybrid MBBR-MBR system and CAS system.

Removal Efficiency	MBBR-MBR	CAS
COD	93%	83%
TSS	99%	66%
Color	85%	55%
TN	100%	100%
TP	100%	100%

**Table 5:** Chromatic coordinates and color differences between fabrics dyed with the treated effluent and the reference dyeing.

100% Effluent Reused	DL_CMC_	DC_CMC_	DH_CMC_	DE_CMC(2:1)_
Yellow Procion HEXL	0.34	−0.38	0.90	1.04
Crimson Procion HEXL	−0.29	−0.39	−0.37	0.61
Navy Procion HEXL	0.38	0.16	−0.24	0.48

**Table 6:** CAS operational cost for treating 1 m^3^ wastewater.

Concept						Total Price €/m^3^	Reference
**(a) Consumption**	**Unit**	**Amount**	**Unit**	**Unit price**	**Convert to** **€/m^3^**	**0.55**	
Electricity	kWh/m^3^	0.96	€/kWh	0.187	0.17952		[44]
Decolorizing agent	kg/m^3^	0.2	€/kg	1.85	0.37		[45]
**(b) Environmental tax**	**Unit**	**Amount**	**Unit**	**Unit price**		**0.86**	
Sludge generation	kg/m^3^	0.83	€/kg	0.158	0.013114		[46]
Wastewater discharge							[10]
OM ^1^	kg/m^3^	0.23	€/kg	1.0023	0.230529		
TSS	kg/m^3^	0.32	€/kg	0.5011	0.160352		
N	kg/m^3^	0.008	€/kg	0.761	0.006088		
P	kg/m^3^	0.003	€/kg	1.5222	0.0045666		
Conductivity	S/cm	0.00598	€/Sm^3^/cm	8.0198	0.0479584		
Summation					0.449494		
ST ^2^ = 1.5 × SUM					0.67424101		
GT ^3^					0.163		
**Total price**						**1.41**	

^1^ OM: organic matter (OM = 2/3COD); ^2^ ST: specific tax; ^3^ GT: general tax.

**Table 7:** MBBR-MBR operational cost for treating 1 m^3^ wastewater.

Concept						Total Price €/m^3^	Reference
**(a) Consumption**	**Unit**	**Amount**	**Unit**	**Unit price**	**Convert to** **€/m^3^**	**0.21**	
Electricity	kWh/m^3^	1.12	€/kWh	0.187	0.20944		[43]
Decolorizing agent	kg/m^3^	0	€/kg	1.85	0		[45]
**(b) Environmental tax**	**Unit**	**Amount**	**Unit**	**Unit price**		**0.35**	
Sludge generation	kg/m^3^	0.023	€/kg	0.158	0.003634		[46]
Wastewater discharge							[10]
OM	kg/m^3^	0.11	€/kg	1.0023	0.110253		
TSS	kg/m^3^	0.006	€/kg	0.5011	0.003006		
N	kg/m^3^	0.007	€/kg	0.761	0.005327		
P	kg/m^3^	0.001	€/kg	1.5222	0.001522		
Conductivity	S/cm	0.00482	€/Sm^3^/cm	8.0198	0.038655		
Summation					0.123742		
ST = 1.5 × SUM					0.185613		
GT					0.163		
**(c) Membrane replacement**						**0.01**	
**(d) Maintenance and repair**						**0.04**	
**Total price**						**0.61**	

**Table 8:** Cash flow (€) assessment for membrane filtration alternative.

Year	Revenues		Total Revenues	Expenditures		Total Expenditures	Net Cash Flow
	Water Recovery	Reduction in Discharge		CAPEX	OPEX		
1	0	0	0	462,403	0	462,403	−462,403
2	87,275	134,029	221,304	0	135,847	135,847	85,457
3	88,497	135,905	224,402	0	137,749	137,749	86,653
4	89,736	137,808	227,544	0	139,677	139,677	87,867
5	90,992	139,737	230,730	0	141,633	141,633	89,097
6	92,266	141,694	233,960	0	143,616	143,616	90,344
7	93,558	143,677	237,235	0	145,626	145,626	91,609
8	94,868	145,689	240,556	0	147,665	147,665	92,891
9	96,196	147,729	243,924	0	149,732	149,732	94,192
10	97,542	149,797	247,339	0	151,829	151,829	95,511
11	98,908	151,894	250,802	0	153,954	153,954	96,848
12	100,293	154,020	254,313	0	156,110	156,110	98,204
13	101,697	156,177	257,874	0	158,295	158,295	99,578
14	103,121	158,363	261,484	0	160,511	160,511	100,972
15	104,564	160,580	265,145	0	162,758	162,758	102,386

**Table 9:** Inventory analysis of the MBBR-MBR treatment.

Processes Included in LCA	MBBR		Unit/FU	Ecoinvent Unit Process
	Input	Output		
COD	2	0.13	kg	
TSS	0.94	0.01	kg	
N	0.055	0.003	kg	
P	0.010	0.001	kg	
Color	700	105	g Pt-co	
Conductivity	6.46	5.42	mS/cm	
Wastewater	1	1	m^3^	
Sludge		0.021	kg	
Decolorizing agent	0		kg	diethylenetriaminepentaacetic acid (DTPA), at plant/RER(Europe) Unit
Electricity	1.12		kWh	Electricity, medium voltage, production ES, at grid/ES(Spain) Unit

**Table 10:** Comparison of environmental impacts of CAS vs. MBBR-MBR: endpoint analysis.

Treatment	Inputs		Human Health (mPt)	Ecosystems (mPt)	Resources (mPt)
CAS	Electricity (kWh/m^3^)	0.96	22.8	1.9	31.8
	Decolorizing agent(kg/m^3^)	0.2	34.4	3.4	81.2
	TOTAL		57.2	5.3	113.0
MBBR-MBR	Electricity (kWh/m^3^)	1.12	26.6	2.2	37.1
	Decolorizing agent(kg/m^3^)	0	0	0	0
	TOTAL		26.6	2.2	37.1

**Table 11:** Comparison of CAS and MBBR-MBR: midpoint analysis.

Impact Category	Unit	CAS	MBBR-MBR	Impact Reduction of MBBR-MBR
Climate change Human Health	kg CO_2_-eq	1.29	0.08	94%
Ozone depletion	kg CFC-11 eq	1.39 × 10^−7^	2.89 × 10^−8^	79%
Human toxicity	kg 1.4-DB eq	0.12	0.03	79%
Photochemical oxidant formation	kg NMVOC	3.89 × 10^−3^	2.30 × 10^−3^	41%
Particulate matter formation	kg PM10 eq	1.95 × 10^−3^	1.40 × 10^−3^	28%
Ionizing radiation	kg U235 eq	0.16	0.11	33%
Terrestrial acidification	kg SO_2_-eq	6.46 × 10^−3^	4.83 × 10^−3^	25%
Freshwater eutrophication	kg P-eq	7.84 × 10^−5^	2.79 × 10^−5^	64%
Marine eutrophication	kg N-eq	2.24 × 10^−3^	1.76 × 10^−4^	92%
Terrestrial ecotoxicity	kg 1.4-DB eq	2.96 × 10^−4^	6.69 × 10^−5^	76%
Freshwater ecotoxicity	kg 1.4-DB eq	7.40 × 10^−3^	1.02 × 10^−4^	99%
Marine ecotoxicity	kg 1.4-DB eq	1.04 × 10^−3^	2.14 × 10^−4^	79%
Agricultural land occupation	m^2^ year	5.51 × 10^−3^	5.22 × 10^−3^	5%
Urban land occupation	m^2^ year	2.16 × 10^−3^	1.79 × 10^−3^	17%
Natural land transformation	m^2^ year	1.25 × 10^−5^	1.10 × 10^−5^	12%
Water depletion	m^3^	1.12 × 10^−2^	3.33 × 10^−3^	70%
Metal depletion	kg 1Fe eq	2.02 × 10^−3^	8.57 × 10^−4^	58%
Fossil depletion	kg oil eq	5.17 × 10^−7^	1.70 × 10^−7^	67%

## Data Availability

The data presented in this study are available on request from the corresponding author. The data is not publicly available because it is licensed by the Ecoinvent database or because it is owned by the textile company Acabats del Bages (Monistrol de Montserrat, Spain).
